# Graphene Nanoplatelets Render Poly(3-Hydroxybutyrate) a Suitable Scaffold to Promote Neuronal Network Development

**DOI:** 10.3389/fnins.2021.731198

**Published:** 2021-09-20

**Authors:** Matteo Moschetta, Martina Chiacchiaretta, Fabrizia Cesca, Ipsita Roy, Athanassia Athanassiou, Fabio Benfenati, Evie L. Papadopoulou, Mattia Bramini

**Affiliations:** ^1^Center for Synaptic Neuroscience and Technologies, Istituto Italiano di Tecnologia, Genova, Italy; ^2^Department of Experimental Medicine, University of Genova, Genova, Italy; ^3^Department of Materials Science and Engineering, Faculty of Engineering, University of Sheffield, Sheffield, United Kingdom; ^4^Smart Materials Group, Istituto Italiano di Tecnologia, Genoa, Italy; ^5^IRCSS, Ospedale Policlinico San Martino, Genova, Italy; ^6^Department of Cell Biology, Faculty of Science, University of Granada, Granada, Spain

**Keywords:** poly(3-hydroxybutyrate), graphene, scaffold, primary neurons, polyhydroxyalkanoates, neural interface

## Abstract

The use of composite biomaterials as innovative bio-friendly neuronal interfaces has been poorly developed so far. Smart strategies to target neuro-pathologies are currently exploiting the mixed and complementary characteristics of composite materials to better design future neural interfaces. Here we present a polymer-based scaffold that has been rendered suitable for primary neurons by embedding graphene nanoplatelets (GnP). In particular, the growth, network formation, and functionality of primary neurons on poly(3-hydroxybutyrate) [P(3HB)] polymer supports functionalized with various concentrations of GnP were explored. After growing primary cortical neurons onto the supports for 14 days, all specimens were found to be biocompatible, revealing physiological growth and maturation of the neuronal network. When network functionality was investigated by whole patch-clamp measurements, pure P(3HB) led to changes in the action potential waveform and reduction in firing frequency, resulting in decreased neuronal excitability. However, the addition of GnP to the polymer matrix restored the electrophysiological parameters to physiological values. Interestingly, a low concentration of graphene was able to promote firing activity at a low level of injected current. The results indicate that the P(3HB)/GnP composites show great potential for electrical interfacing with primary neurons to eventually target central nervous system disorders.

## Introduction

Smart neuronal interfaces are emerging as promising tools for neural tissue engineering, where scaffolds are required to provide cell support and stimulate network excitability (Lizarraga-Valderrama et al., 2019). Hence, the dynamic relation between neurons and scaffolds can be used to deliver specific stimuli to the cells in order to foster regeneration and tissue repair. This is a crucial aspect in the case of the central nervous system, which has a limited capacity of self-repair and regeneration (Tam et al., 2014). Recently, it has been shown that electrical stimulation of neurons enhances neurite outgrowth as well as neuronal migration, proliferation, and cellular functions (Koppes et al., 2016; Zhang et al., 2018; Chen et al., 2019). In this scenario, the research of innovative electroactive biomaterials able to promote neuronal responses, such as adhesion, proliferation, and network functionality, and potentially deliver electrical stimulation is of utmost interest.

In the last decade, graphene has shown encouraging performances as a neuronal interfacing material because of its exceptional mechanical strength and electrical conductivity (Wick et al., 2014; Santos et al., 2021). These properties, in conjunction with its biocompatibility, have made it a good candidate with high prospects in tissue engineering, especially neural tissue engineering (Bramini et al., 2018). In particular, graphene substrates can sustain neural cultures without changing the neuronal morphology and enhancing the neurite number and length (Li et al., 2011; Convertino et al., 2018, 2020; Capasso et al., 2020; Moschetta et al., 2021). Furthermore, graphene is able to tune neuronal excitability by altering the extracellular environment in an astrocyte-like fashion (Kitko et al., 2018; Pampaloni et al., 2018). More specifically, although the electrical conductivity of graphene *per se* does not seem to significantly affect the adhesion and maturation of neuronal cultures (Capasso et al., 2020), graphene has been shown to interfere with the excitatory and inhibitory synaptic transmission as well as to alter Ca^2+^ signaling and membrane cholesterol composition (Bramini et al., 2016, 2019; Fabbro et al., 2016; Rauti et al., 2016, 2019; Chiacchiaretta et al., 2018; Durso et al., 2018; Kitko et al., 2018; Pampaloni et al., 2018).

However, most of these studies have been performed using graphene flakes in solution or chemical vapor deposition (CVD) graphene films which, despite their high quality, are both very difficult to handle due to flake hydrophobicity (that leads to aggregation) and residual metal contaminants in the CVD preparation (that can lead to cytotoxic effects) (Firme and Bandaru, 2010). To overcome these issues, an alternative approach in the context of smart neuronal interfaces is to use graphene as a filler in biocompatible polymer matrices (Shin et al., 2016; Golafshan et al., 2017; Ma et al., 2019).

A class of biomaterials that is gaining a lot of attention are polyhydroxyalkanoates (PHAs). PHAs are natural linear polyesters produced by microorganisms, mainly bacteria, that are biodegradable and highly biocompatible (Sosa-Hernández et al., 2020). The physical properties of PHAs depend on the microbial strain used, the carbon source, and the extraction method, making PHAs highly versatile. Because of their characteristics, PHAs can be used in a wide range of applications, spanning from food packaging to tissue engineering (Sosa-Hernández et al., 2020). The most widely used and characterized PHA is poly(3-hydroxybutyrate) or P(3HB). P(3HB) exhibits excellent biocompatibility and biodegradability and has already been investigated in tissue engineering applications (Lizarraga-Valderrama et al., 2016) as cardiac (Malm et al., 1992) and bone (Mohamad Yunos et al., 2008) tissue engineering scaffolds. Only a few works have investigated the biological effects of P(3HB) on neural tissue, e.g., in neural stem cell growth (Xu et al., 2010), peripheral nerve regeneration (Hazari et al., 1999; Hinüber et al., 2014; Lizarraga-Valderrama et al., 2015, 2020), or neuron-like cell alignment (Lizarraga-Valderrama et al., 2019), without addressing other biological effects when directly interfacing primary neurons. In addition, P(3HB) is stiff, brittle, and crystalline, characteristics that are not always the best fit for cellular interfacing (Veliev et al., 2016). In most cases, biomedical applications, at large, and tissue engineering, in particular, require the polymer properties to be adjusted by changing the composition from a single material to multiple materials (Iqbal et al., 2015a,b,c).

Herein we have prepared P(3HB) composites with graphene nanoplatelets (GnP) with the aim of rendering such polymer conductive, thus more appropriate and appealing for neurobiology applications. We have investigated P(3HB)/GnP biocompatibility and electrophysiological properties, for different GnP concentrations, in primary neurons. Our strategy was to prepare P(3HB)/GnP composite islands on PET supports, resulting in uncovered PET areas with uncompromised transparency, that could facilitate the electrophysiological investigation of the neuronal network grown on the composite. To the best of our knowledge, this is the first report to investigate the functionality of neuronal networks onto graphene composites through their electrophysiological activity. Practical difficulties that come from the opacity of the polymeric composites have rendered such investigations a very difficult task with biopolymers, and data are still missing from the respective literature (Park et al., 2010; Kuzum et al., 2014; Feng et al., 2015; Genchi et al., 2015; Aznar-Cervantes et al., 2017; Golafshan et al., 2017; Bei et al., 2019; Ma et al., 2019). In the present research, although cell viability experiments show good viability for both pure P(3HB) and P(3HB)/GnP, electrophysiology experiments display that primary neurons grown on pure P(3HB) do not reach physiological values in terms of firing properties (action potentials), in contrast to neurons grown on conductive P(3HB)/GnP composites. The importance of this result lies on the realization that standard live–dead assays do not reflect the correct development of the functionality of a network, thus making electrophysiology studies a must-do once exploring neuronal activity in an unfamiliar environment.

## Materials and Methods

### Sample Preparation

Graphene nanoplatelets (GnP, grade Ultra-G+) were provided by Directa Plus Spa (Italy). The lateral dimension of the platelets is a few tens of micrometers, while their thickness is a few tens of nanometers. Chloroform was acquired from Sigma-Aldrich and used as received. P(3HB) was first dissolved in chloroform to result in 5 wt% of polymer solution. Subsequently, GnP were added, with concentrations ranging from 0 to 10 wt%, and bath-sonicated at 59 Hz for 7 h so that a homogeneous dispersion of the GnP fillers formed in the polymer solution. The islands on the PET substrate were made by drop-casting 20–40 μl of the composite solution on PET on different non-geometrically defined locations. Subsequently, they were covered to allow slow evaporation, resulting in composite islands on the PET, with a diameter of ∼1 cm and thickness of 40–50 μm. Alternatively, the composite solution was cast on glass petri dishes, and the solvent was allowed to evaporate, resulting in free standing films. All composites were prepared under constant ambient conditions.

### Scanning Electron Microscopy

The morphology of the films and of the neurons was studied by scanning electron microscopy (SEM, JEOL JSM-6490LA) after they had been coated by a thin gold layer in order to improve the electrical conductivity. For SEM cell analysis, primary cortical neurons cultured for 14 days *in vitro* were fixed with 1.5% glutaraldehyde in 66 mM sodium cacodylate buffer and postfixed in 1% OsO_4_. Sample dehydration was performed by 5 min washes in 30, 50, 70, 80, 90, 96, and 100% EtOH solutions, followed by overnight incubation at 100% EtOH.

### Thermal Characterization

The degradation temperature of the materials was evaluated by thermogravimetric analysis (TGA). During the TGA measurements, the samples were heated from 30 to 800°C at a heating rate of 10°C/min under nitrogen atmosphere set at a flow rate of 50 ml/min. TGA was performed on free-standing films.

### Raman Spectroscopy

Micro-Raman spectra were collected at ambient conditions using a Horiba Jobin Yvon LabRAM HR800 μRaman spectrometer equipped with a microscope. A 632.8-nm excitation line, in backscattering geometry through a × 50 objective lens, was used to excite the specimens at low power of ca. 0.25 mW. The experimental setup consists of a grating of 600 lines per millimeter with a spectral resolution of approximately 1 cm^–1^.

### Preparation of Primary Neurons

All experiments were carried out in accordance with the guidelines established by the European Community Council (Directive 2010/63/EU of 22 September 2010) and were approved by the Italian Ministry of Health. Primary cortical cultures were prepared from wild-type C57B6J mice (Charles River, Calco, Italy). All efforts were made to minimize suffering and to reduce the number of animals used. The mice were sacrificed by CO_2_ inhalation, and 18-day embryos were removed immediately by cesarean section. Briefly, enzymatically dissociated cortical neurons were plated on poly-D-lysine-coated (0.1 mg/ml) PET glass coverslips (Thermo-Fischer Scientific, Waltham, MA), PET-Gr, and PET-HGr supports at a density of 30,000 cells/cm^2^. The cultures were incubated at 37°C, 5% CO_2_, and 90% humidity in a medium consisting of Neurobasal (Gibco/Thermo-Fischer Scientific) supplemented to reach a final concentration of 5% L-glutamine (200 mM), 5% penicillin (10,000 units/ml)/streptomycin (10,000 μg/ml), and 10% B27 supplement (Gibco/Thermo Fischer Scientific). Following this protocol, astrocyte presence was previously reported to be less than 6% of the total number of cells (Bramini et al., 2016). To avoid astrocyte proliferation, the cells were plated and allowed to adhere in the absence of supplements (i.e., fetal bovine serum) that could promote glial cell proliferation. All chemicals were purchased from Life Technologies/Thermo-Fischer Scientific unless stated otherwise.

### Cell Viability

**FIGURE 1:**
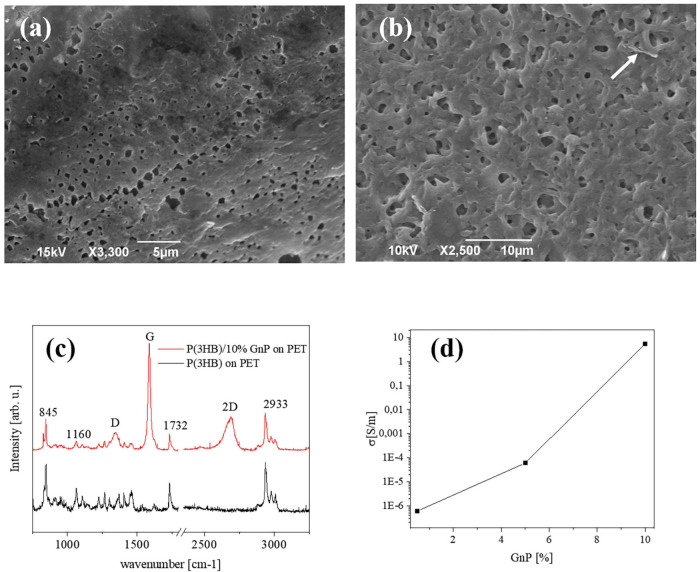
Typical SEM images of the surface of **(a)** pure P(3HB) films and **(b)** P(3HB) film containing 10 wt% GnP. **(c)** μRaman spectra of P(3HB) and composites **(d)** electrical conductivity of the composite islands deposited on PET, versus the GnP concentration.

Mouse cortical neurons were seeded onto P(3HB) supports with different GnP concentrations (0.5, 3.0, and 10.0% GnP) for 14 days. For clarity, we considered two different control conditions: glass, the traditional support for primary neuron growth, and P(3HB). Live cells were stained with propidium iodide (1 μM) for cell death quantification, fluorescein diacetate (2 μM) for cell viability, and Hoechst 33342 (1 μM) for nuclei visualization for 3 min at room temperature (RT). Cell viability was quantified at × 20 (0.5 NA) magnification using a Nikon Eclipse-80i upright epifluorescence microscope (Nikon, Tokyo, Japan), with random sampling of 10 fields per sample (*n* = 2 samples from two independent culture preparations). The percentage of propidium iodide (PI)-positive cells with respect to the total number of Hoechst-positive cells was calculated for each experimental group and normalized to the values of the untreated samples, set to 1 (black line in Figure 1b). Image analysis was performed using the ImageJ software and the Cell Counter plugin.

### Immunofluorescence Staining and Confocal Laser Scanning Microscopy

Mouse cortical neurons were fixed in phosphate-buffered saline (PBS)/4% paraformaldehyde for 20 min at RT. The cells were permeabilized with 1% Triton X-100 for 5 min, blocked with 2% fetal bovine serum in PBS/Tween 80 0.05% for 30 min at RT, and incubated with mouse monoclonal anti-β-tubulin III antibodies (#T2200, Sigma-Aldrich) in the same buffer for 45 min. After the primary incubation and several PBS washes, the neurons were incubated for 45 min with fluorescently conjugated secondary antibody (Alexa Fluor 488, #A11029; Thermo-Fisher Scientific) in a blocking buffer solution. The samples were mounted in ProLong Gold antifade reagent with DAPI (#P36935, Thermo-Fisher Scientific) on 1.5-mm-thick coverslips. Image acquisitions were performed using a confocal microscope (SP8, Leica Microsystems GmbH, Wetzlar, Germany) at × 40 (1.4 NA) magnification.

### Patch-Clamp Recordings

Mouse cortical neurons were taken from C57B6J mice and recorded at 14 DIV. Patch pipettes, prepared from thin borosilicate glass, were pulled and fire-polished to a final resistance of 4–5 MΩ when filled with the standard internal solution. Current-clamp recordings were performed in morphologically identified pyramidal neurons at a holding potential of -70 mV. Action potential (AP) firing was induced by injecting current steps of 10 pA lasting for 500 ms, acquired at 50 kHz and filtered at 1/5 of the acquisition rate with a low-pass Bessel filter. The mean firing frequency was calculated as the number of APs evoked by a minimal current injection in 500 ms, whereas the instantaneous frequency was estimated as the reciprocal value of the time difference between the first two evoked APs. Data acquisition was performed using the PatchMaster program (HEKA Elektronic), and data analysis was performed with the Prism software (GraphPad Software, Inc.). The shape properties of the first AP elicited by a minimal current injection were analyzed by building time derivatives of voltage (d*V*/d*t*) vs. voltage plots (phase–plane plots) as previously described (Valente et al., 2016; Prestigio et al., 2019). The phase–plane plots were obtained and analyzed with the software OriginPro-8 (OriginLab Corp., Northhampton, MA, United States). All experiments were performed at room temperature. For all the experiments, the cells were maintained in extracellular standard solution (Tyrode) containing the following (in mM): 140 NaCl, 2 CaCl_2_, 1 MgCl_2_, 4 KCl, 10 glucose, and 10 HEPES (pH 7.3 with NaOH), in which D-(-)-2-amino-5-phosphonopentanoic acid (50 μM), 6-cyano-7 nitroquinoxaline-2, 3-dione (10 μM), bicuculline methiodide (30 μM), and (2S)-3-[[(1S)-1-(3,4-dichlorophenyl)ethyl]amino-2-hydroxypropyl] (phenylmethyl) phosphinic acid hydrochloride (5 μM) were added to block the NMDA, non-NMDA, GABA A, and GABA B receptors, respectively. The internal solution (K-gluconate) was composed of (in mM) 126 K gluconate, 4 NaCl, 1 MgSO_4_, 0.02 CaCl_2_, 0.1 BAPTA, 15 glucose, 5 HEPES, 3 ATP, and 0.1 GTP (pH 7.3 with KOH). All the reagents were bought from Tocris, unless otherwise specified in detail.

### Statistical Analysis

All data are expressed as mean ± standard errors of the mean (SEM) or standard deviation (SD) for the number of cells (*n*) or independent preparations, as detailed in the figure legends. Normal distribution of data was assessed using the D’Agostino–Pearson normality test. To compare more than two normally distributed experimental groups, one-way ANOVA (followed by Bonferroni’s multiple-comparison test) was used. To compare more than two experimental groups that are not normally distributed, the Kruskal–Wallis test was used, followed by Dunn’s multiple-comparison test. Significance level was preset to *p* < 0.05. Statistical analysis was carried out using Prism (GraphPad Software, Inc., La Jolla, CA).

## Results

**SCHEME 1:**
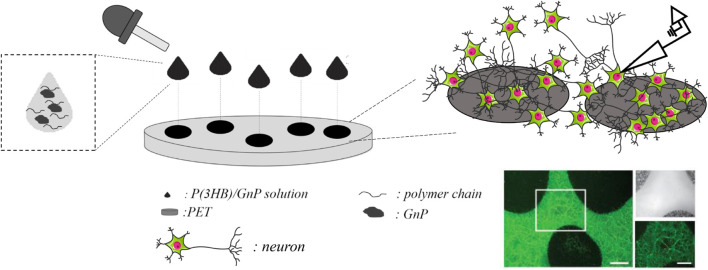
Deposition of the P(3HB)/GnP composite islands on PET and their interface with neurons; representative image of a neuronal culture, stained with fluorescein diacetate (in green) for viable cells. Scale bar: 200 μm in the main image and 100 μm in the zoomed images.

The preparation method of the P(3HB)/GnP island deposition on the PET substrate and their interface with primary neuron cultures is schematically described in Scheme 1.

Briefly, 20–40 μl droplets of P(3HB)/GnP composite solution were randomly deposited on PET so that the droplets did not coalesce and create a patchwork of covered and uncovered areas that allowed light transmission and facilitated electrophysiology measurements. The diameter of the islands was ∼1 cm, and their thickness was in the range of 40–50 μm.

The morphological investigation shown in Figure 1a depicts the surface of the P(3HB) islands on PET, presenting the rough structure that is typically seen in free-standing films of the same material (Misra et al., 2008; Papadopoulou et al., 2019). Upon addition of GnP to the P(3HB) matrix, the surface attains a slightly rougher morphology, and the GnP are generally not exposed on the surface of the polymer. A representative image is shown in Figure 1b for 10 wt% GnP. Thermal degradation analysis (performed on free-standing films), presented in Supplementary Figure 1, shows that the decomposition of P(3HB) starts at 260°C and a gradual loss of the composite material resulting in an increasing amount of remaining material, i.e., GnP. The Young’s modulus of pure P(3HB) is about 1.2 GPa, while upon the addition of GnP it decreases, reaching to about 0.7 GPa for 10 wt% GnP (Papadopoulou et al., 2019).

Figure 1c displays a typical μRaman spectra from pure P(3HB) and P(3HB)/GnP (10% w/w) island on PET. All spectra exhibit the main vibrational modes of P(3HB) at 840 cm^–1^ (C–COO stretching), 1,060 cm^–1^ (C–CH_3_ stretching), 1,223, and 1,264 cm^–1^ [helical structure of crystalline P(3HB)], 1,726 cm^–1^ (C = O stretching), a split band at 1,445/1,457 cm^–1^ (band splitting seen in polymers with helical structures due to intermolecular interactions), and several peaks within the region 2,800–3,100 cm^–1^ (C–H stretching from the methyl, methylene, and methene groups). In the case of the P(3HB)/GnP composites, the fingerprint peaks of GnP appear at 1,340 cm^–1^ (D peak), 1,582 cm^–1^ (G peak), and 2,686 cm^–1^ (2D peak) (Papadopoulou et al., 2019).

Finally, the electrical transport properties of the composite islands on PET are shown in Figure 1d. In accordance with our previous work (Papadopoulou et al., 2019), the electrical conductivity of the initially insulating P(3HB) increases upon the addition of GnP, reaching approximately 5 S/m for 10% w/w GnP. More extensive characterization on the pure P(3HB) and P(3HB)/GnP is presented in our previous work (Papadopoulou et al., 2019).

**FIGURE 2:**
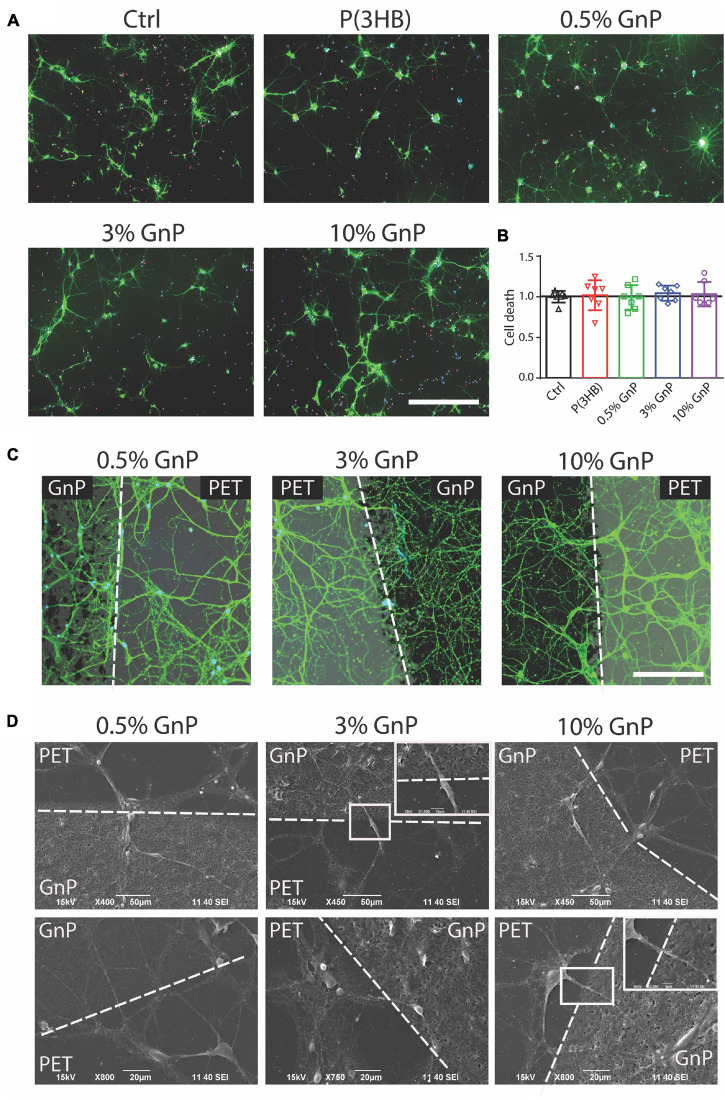
Neuronal viability on P(3HB)/GnP substrates. Primary mouse cortical neurons were grown for 14 days onto glass, P(3HB), or P(3HB) containing 0.5, 3.0, and 10.0% GnP, respectively. **(A)** Representative images of a neuronal culture, stained with Hoechst-33342 (blue) for nuclei visualization, fluorescein diacetate (FDA, in green) for cell viability, and propidium iodide (PI) for dead cell visualization (scale bar: 200 μm). **(B)** Cell viability, evaluated by fluorescence microscopy, is shown. The percentages of propidium iodide (PI)-positive cells with respect to the total number of Hoechst-positive cells, calculated for each experimental group, were normalized to the values of glass substrate set to 1. No significant changes in cell death were observed under all the experimental conditions (data are expressed as mean ± SD. One-way ANOVA/Bonferroni’s tests, *n* = 7 fields per experimental condition, from two independent neuronal preparations). **(C)** High magnification of neuronal cultures grown onto P(3HB)/GnP composites and stained by FDA (white dashed lines indicate the GnP island borders. Scale bar: 20 μm). **(D)** SEM micrographs and relative higher magnifications of neuronal networks grown onto P(3HB)/GnP composites. Cells were able to adhere and properly grow across the PET support and the islands.

Mouse cortical neurons were then grown onto the P(3HB)/GnP islands for 14 days, as shown in Figure 2A and Supplementary Figures 2, 3. At this point, cell viability studies, as well as electrophysiology investigations, were performed in order to assess network wellness and functionality. The first step, as displayed in Figure 2B, was the evaluation of cell death at 14 days of culture onto the various samples, including glass and P(3HB) controls. The cells were live-stained with fluorescein diacetate (FDA), PI, and Hoechst-33342 to identify live cells, dead cells, and all cell nuclei, respectively. The results show that no cell death was observed and all scaffolds presented a good biocompatibility with the primary neurons, in accordance with previous results on pure P(3HB) (Novikov et al., 2002; Xu et al., 2010).

The network morphology was then investigated by both confocal laser scanning microscope (CLSM) and SEM. In Figures 2C,D and Supplementary Figure 3, the images acquired from CLSM and SEM display a rich neuronal growth (green network) on and in between P(3HB)/GnP islands (black spots). The acquired images highlight the physiological morphology and organization of the neuronal network onto all the tested supports in the absence of cell clusters or aggregates, supporting the high biocompatibility of the scaffolds. The neuronal network spread all over the scaffold, showing cells crossing the edge of P(3HB)/GnP islands, testifying the excellent properties of the interface.

**FIGURE 3:**
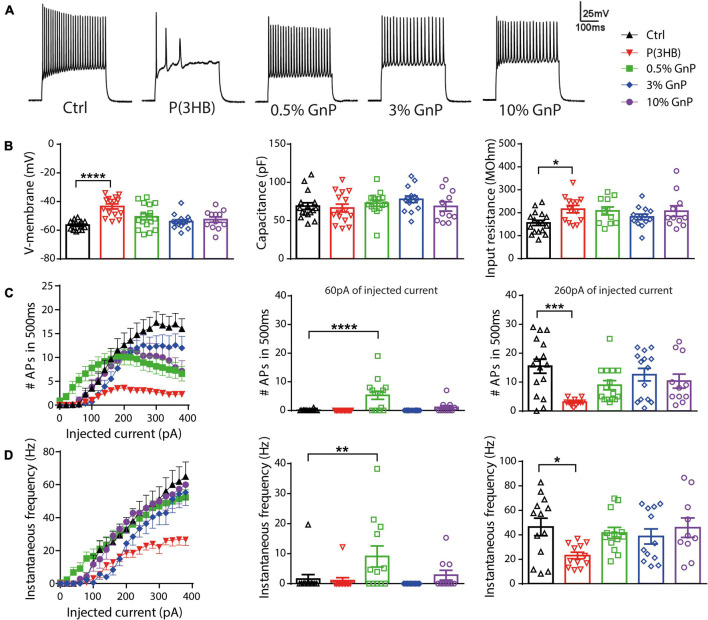
Firing properties of primary cortical neurons plated on P(3HB)/GnP composites. Neurons were plated at increasing GnP concentrations (0.5, 3.0, and 10.0% GnP) and under two control conditions [glass and P(3HB)] for 14 days. **(A)** Representative whole-cell current-clamp traces of action potentials (APs) evoked by the injection of 260 pA current step are shown. **(B)** Resting membrane potential [left, *n* = 17, 15, 16, 14, and 12 for Ctrl, P(3HB), 0.5, 3.0, and 10.0% GnP, respectively], capacitance [center, *n* = 16, 15, 15, 14, and 11 for Ctrl, P(3HB), 0.5, 3.0, and 10.0% GnP, respectively], and input resistance [right, *n* = 17, 13, 11, 13, and 11 for Ctrl, P(3HB), 0.5, 3.0, and 10.0% GnP, respectively] are shown. **(C)** The mean number of APs in 500 ms as a function of the injected current (left) and the mean firing frequency at 60 pA (center) and 260 pA (right) injected current are shown [*n* = 16, 13, 15, 14, and 11 for Ctrl, P(3HB), 0.5, 3.0, and 10.0% GnP, respectively]. **(D)** Instantaneous frequency as a function of the injected current (left) and at 60 pA (center) and 260 pA (right) injected current are shown [*n* = 13, 12, 13, 13, and 10 for Ctrl, P(3HB), 0.5, 3.0, and 10.0% GnP, respectively]. 0.5% GnP displayed a significant enhancement in both instantaneous and mean frequency compared to the glass controls. In addition, no significant differences between composite samples and glass controls were found. On the contrary, neurons plated on the P(3HB) support showed a reduced frequency of evoked APs. Data are expressed as means ± SEM; Kruskal-Wallis and Dunn’s *post hoc* tests, from two independent neuronal preparations. *p < 0.05; **p < 0.01; ***p < 0.001; ****p < 0.0001.

Furthermore, cell viability and electrophysiology recordings were also tested and performed for cortical neurons grown onto free-standing films, as reported in the (Supplementary Figures 4, 5). However, cell viability was compromised with an increasing percentage of GnP, and because of the opacity of the films, we were not able to carry out electrophysiology studies in live neurons. Thus, the new composite island design prepared onto transparent PET supports has clearly shown its potential and advantages compared to P(3HB)/GnP free-standing films when it comes to primary neuron bio-interaction (Scheme 1). To assess neuronal network formation and maturation, electrophysiology investigations were extensively carried out *via* whole-cell current-clamp recordings (Figure 3). Single neuron intrinsic excitability was measured as a parameter of neuronal culture wellness. Action potentials were evoked by injecting increasing steps of current, and the passive (membrane capacitance, input resistance, and resting potential) and active firing properties were evaluated (Figure 3A).

Interestingly, the resting membrane potential of neurons plated on pure P(3HB) support was shifted to more positive values, resulting in a strong cell membrane depolarization with a concomitant increase in cell input resistance. Despite these alterations in passive membrane properties, neurons grown onto P(3HB) showed a membrane capacitance comparable to the control cells (Figure 3B). On the contrary, neurons plated onto P(3HB) with 0.5, 3.0, or 10.0% GnP showed similar passive membrane properties to neurons grown onto traditional glass supports. These data suggest that the presence of GnP (independent of its concentration and electrical conductivity) is sufficient to restore the physiological membrane passive properties affected by P(3HB). In order to investigate if the alterations in passive membrane properties result in changes in neuronal intrinsic excitability, the capability of primary neurons to generate trains of APs was evaluated by calculating the mean firing frequency and the instantaneous firing frequency (Figures 3C,D). The AP generation is a proxy of the capability of the supports to guarantee proper neuronal maturation and functionality. Neurons were clamped at a holding potential of −70 mV, and APs were evoked by the injection of steps of increasing current. Mean and instantaneous firing frequencies were calculated as described in section “Materials and Methods.” In accordance with the passive membrane property results, cortical neurons grown onto pure P(3HB) presented a significant reduction of both mean and instantaneous AP frequency compared to cells grown onto glass coverslips. Although viability tests did not report any toxic effect of the materials (Figure 1b), they are not *per se* sufficient to ensure normal neuronal network functionality and proper cellular physiology. Indeed through an electrophysiology investigation, we show that neurons plated on the GnP/P(3HB) composites display, in general, a firing frequency comparable to neurons grown onto traditional glass controls, indicating that the presence of GnP was responsible for the restoration of the physiological network activity. Interestingly, neurons grown onto 0.5% GnP showed a remarkable increase in both mean and instantaneous frequency at 60 pA of injected current when compared to neurons grown onto glass control. On the contrary, no significant differences were reported among neurons grown onto the other composites. A hypothesis to explain the observations could be that the high electrical conductivity of graphene could improve neuronal activity and communication. Indeed numerous recent studies (Tang et al., 2013; Fabbro et al., 2016; He et al., 2016) attributed the reported alteration on cell excitability and/or an enhancement in synaptic transmission strength to graphene electrical conductivity by speculating that graphene films lead to a change in cation channel composition (in particular, K^+^ and Na^+^ channels). Our results indicate that a crucial parameter, in order to promote the normal electrophysiological activity of the neuronal network, is the electrical properties of the scaffold, for a wide range of electrical conductivity values. This is in accordance with a recent study, where it is clearly shown that neurons plated on CVD graphene sheets with a different electrical conductivity maintain the physiological values of the membrane passive properties independent of the value of the electrical conductivity (Capasso et al., 2020). Indeed a fully coated conductive film is not necessary, but even single conductive spots are sufficient to restore the physiological neuronal properties.

**FIGURE 4:**
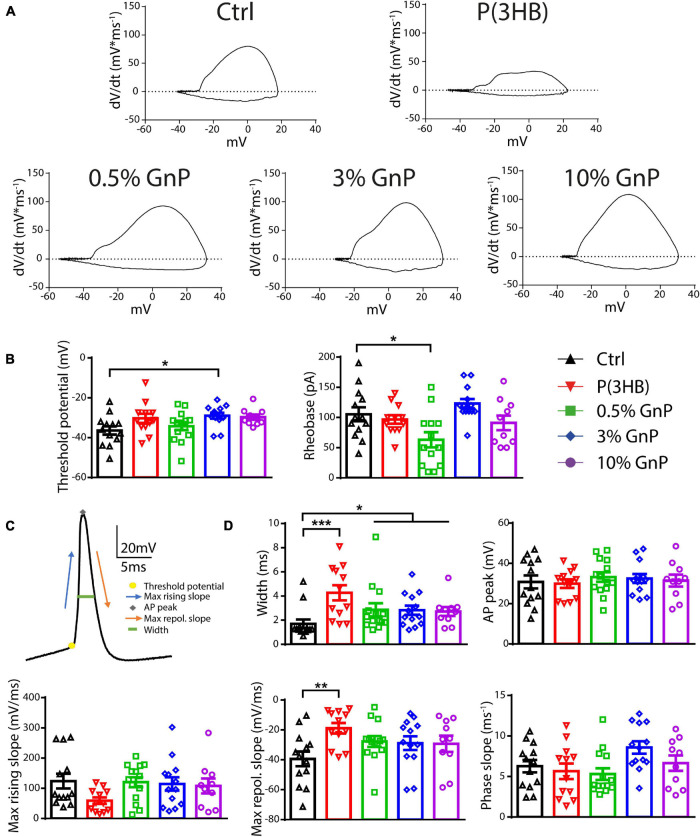
Passive and active properties of primary cortical neurons plated upon P(3HB)/GnP composites. Neurons were plated in various GnP concentrations (0.5, 3.0, and 10.0% GnP) and two control conditions [glass and P(3HB)] for 14 days. **(A)** Representative phase-plane plots of the first action potential in the train are shown. **(B)** Threshold potential (left) and rheobase (right) are shown. **(C)** Representative shape of the first AP showing its main parameters. **(D)** Width (upper panel, left), AP peak (upper panel, right), maximum rising slope (lower panel, left), maximum repolarizing slope (lower panel, center), and phase slope (lower panel, right) are shown. Cortical neurons plated onto GnP supports displayed a small but significant enhancement in the AP width; in addition, cortical neurons plated on P(3HB) supports showed an increased width and maximum repolarizing slope. All data are expressed as means ± SEM; one-way ANOVA/Kruskal-Wallis test and Dunn’s *post hoc* test, *n* = 13, 12, 14, 13, and 10 for Ctrl, P(3HB), 0.5, 3.0, and 10.0% GnP, respectively, from two independent neuronal preparations. *p < 0.05; **p < 0.01; ***p < 0.001.

To clarify the mechanism by which the reduction in AP firing of neurons grown onto P(3HB) substrates occurred, we evaluated the AP shape parameters by calculating the membrane potential change rate (the first derivative d*V*/d*t*) vs. voltage, known as phase–plane plot (Figure 4). Phase–plane plots traditionally exhibit two components during AP generation (Figures 4A,C). The first component is characterized by a voltage increase from the baseline due to the AP initiation at the level of the axon initial segment. The second component is generated when the AP invades the cell soma and increases until it reaches the AP peak. This analysis permits a better resolution of the AP kinetics, directly dependent on voltage-gated Na^+^ channel conductance (McCormick et al., 2007; Shu et al., 2007; Yu et al., 2008). In detail, we considered the following: (i) the AP peak (the positive voltage value at 0 d*V*/d*t*) that represents the maximum depolarization, (ii) the AP width as the distance between the depolarizing and repolarizing phases at 0 mV, (iii) the threshold potential as the potential at which the AP is triggered, and (iv) the rheobase as the current needed to evoke an AP. Cortical neurons, plated onto P(3HB) supports, displayed a strong enhancement of AP width. This data suggests a possible alteration in the AP dynamics since the maximum AP peak is not changed (Figures 4B–D). The significant alterations in AP dynamics and the consequent increase in the AP width could be at the base of the reduction in the mean and instantaneous frequency. Neurons plated onto 0.5% GnP showed a significant decrease of the rheobase and a consequent reduction of the amount of current needed to generate APs. In fact, neurons grown onto 0.5% GnP displayed an enhancement of the firing frequency at 60 pA of injected current. Interestingly, neurons grown onto 3 and 10% GnP did not show changes in both rheobase and firing rate, suggesting that the increasing residual conductivity does not affect these parameters, in accordance with Capasso et al. (2020). Interestingly, neurons grown onto all GnP/P(3HB) composites showed a small but significant increase in width together with a significant increase in threshold voltage that was found in neurons plated onto 3.0% GnP when compared to neurons grown onto the control glass (Figures 4B–D). Next, the maximum rising slope, the maximum repolarizing slope, and the phase slope were measured. An increase in the maximum repolarizing slope and a slight reduction in the maximum rising slope were observed in neurons grown onto pure P(3HB); as expected, these values were normalized in neurons grown onto GnP/P(3HB) composites (Figure 4D). The increase in width and repolarizing slope observed in neurons plated onto P(3HB) might be at the base of the observed reduction in firing frequency. Altogether these data suggest that, despite its biocompatibility, P(3HB) does not *per se* promote proper neuronal network maturation in terms of functionality. However, the addition of GnP can rescue the alterations in firing properties induced by P(3HB) by providing a suitable electrical environment. In addition, composites with a low concentration of graphene are able to boost the firing activity by promoting AP occurrence already at a low level of injected current.

## Discussion and Conclusion

In the last decade, different neuronal interfaces have been developed for nerve repair/regeneration and functional recovery after neuronal injury; among these, recently, graphene-based materials have begun to be investigated due to their attractive physico-chemical properties (Akhavan, 2016; Bei et al., 2019). Here we have developed a P(3HB) polymer matrix with embedded GnP that results in the increase of matrix electrical conductivity. The composite P(3HB)/GnP promoted neuronal growth and maturation on the composite islands as well as in the space between.

The proposed scaffolds are innovative in the island design, which stresses the concept of minimally modifying a polymer without fully coating/functionalizing it. The presence of few conductive “hot-spots” arranged onto the PET support is enough to significantly improve the neuronal network interactions. First of all, our supports displayed excellent biocompatibility, and cortical neurons were able to rapidly adhere and wire at all GnP concentrations tested in a way comparable with neurons plated on glass. Neurons grown onto pure P(3HB) showed impaired firing frequency; however, the presence of GnP was able to rescue the firing property alterations to physiological levels. Since the topography and surface roughness of the islands is very similar among all samples tested, it is reasonable to conclude that the presence of GnP, by rendering the P(3HB) conductive, is responsible for the recovery of physiological cell signaling. Nonetheless, a small concentration of GnP (0.5%) is sufficient to improve neuron activity, providing a suitable electrical environment and thus triggering proper neuronal network formation. Of note is that our results suggest that a small residual conductivity of the scaffold is sufficient (together with high biocompatibility and good cell adhesion) to see a significant effect in neuronal network development compared to non-conductive supports. On the contrary, highly conductive graphene-based interfaces promote, without significantly improving, neuronal activity in a more physiological manner (Capasso et al., 2020). The search for highly conductive neuronal interfaces might not be as crucial as thought so far.

In summary, in this work, we were able to transform a less responsive biopolymer like P(3HB) into a promising neuronal bio-interface by embedding GnP at low concentrations. We also proved that primary cortical neurons plated on P(3HB)/GnP islands displayed an electrophysiological behavior comparable with neurons plated on glass, paving the way to composite scaffolds for efficient neuronal interfaces.

## Data Availability Statement

The original contributions presented in the study are included in the article/Supplementary Material, further inquiries can be directed to the corresponding author/s.

## Author Contributions

EP, MB, FC, AA, and FB conceptualized and supervised the project. IR provided the P(3HB) biopolymer. EP prepared and characterized the composites. MB performed neuron cultures, SEM imaging, and cell viability assay. MM and MC performed and analyzed the electrophysiological experiments. MM, EP, and MB wrote the manuscript. All authors contributed to the article and approved the submitted version.

## Conflict of Interest

The authors declare that the research was conducted in the absence of any commercial or financial relationships that could be construed as a potential conflict of interest.

## Publisher’s Note

All claims expressed in this article are solely those of the authors and do not necessarily represent those of their affiliated organizations, or those of the publisher, the editors and the reviewers. Any product that may be evaluated in this article, or claim that may be made by its manufacturer, is not guaranteed or endorsed by the publisher.
