# Supplementary material for: Graphene Nanoplatelets Render Poly(3-Hydroxybutyrate) a Suitable Scaffold to Promote Neuronal Network Development
Source: Front Neurosci. 2021 Sep 20;15:731198. doi: 10.3389/fnins.2021.731198 (PMC8488094; doi:10.3389/fnins.2021.731198)
Supplement: Supplementary file 1 [file Data_Sheet_1.pdf]

Supplementary Material

Graphene nanoplatelets render poly(3-hydroxybutyrate) a suitable scaffold to promote neuronal network development

Matteo Moschetta[1,2,*], Martina Chiacchiaretta[1,Δ], Fabrizia Cesca[1,Σ], Ipsita Roy,[3] Athanassia Athanassiou[4], Fabio Benfenati[1,5], Evie L. Papadopoulou[4,#,*] and Mattia Bramini[1,6,#,*]

[1] Center for Synaptic Neuroscience and Technologies, Istituto Italiano di Tecnologia, L. go Rosanna Benzi 10, 16132 Genova, Italy

[2] Department of Experimental Medicine, University of Genova, Viale Benedetto XV, 16132 Genova, Italy

[3] Applied Biotechnology Research Group, School of Life Sciences, College of Liberal Arts and Sciences, University of Westminster, London W1W 6UW, UK

[4] Smart Materials, Istituto Italiano di Tecnologia, via Morego 30, 16163 Genoa, Italy

[5] IRCSS, Ospedale Policlinico San Martino, L. go Rosanna Benzi 10, 16132 Genova, Italy

[6] Department of Cell Biology, Faculty of Science, University of Granada, 18071 Granada, Spain

[Δ] Present Address: Department of Neuroscience, Tufts University School of Medicine, Boston, MA 02111, USA

[Σ] Present Address: Department of Life Sciences University of Trieste, 34127 Trieste, Italy

***Correspondence:**
Matteo Moschetta,
E-mail: matteo.moschetta@iit.it
Evie L. Papadopoulou,
E-mail: paraskevi.papadopoulou@iit.it
Mattia Bramini,
E-mail: mbramini@ugr.es

[#] These authors contributed equally

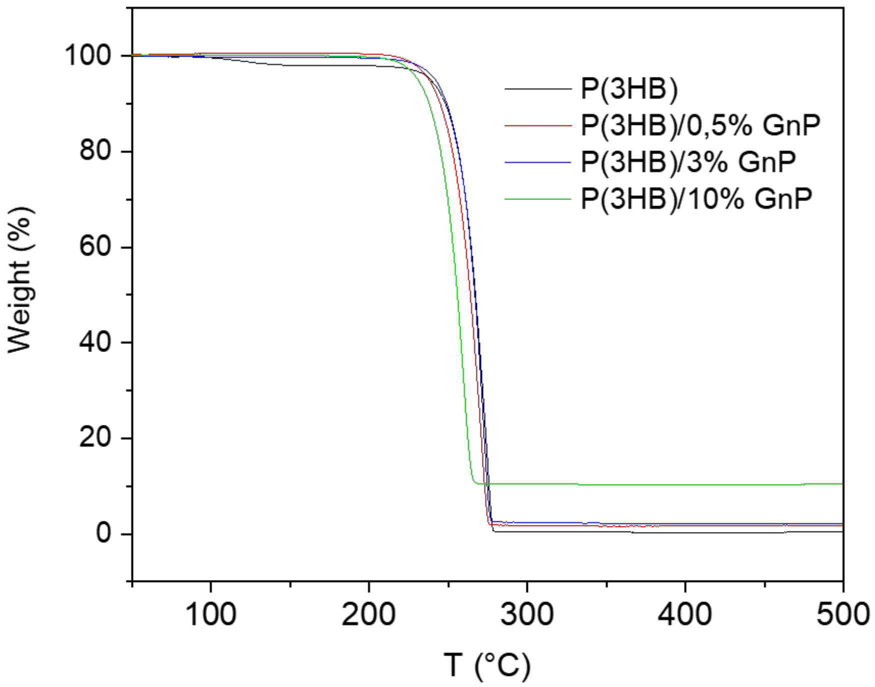

Figure S1: Thermal degradation analysis of P(3HB)/GnP composites.

Thermographs presented in Figure S1, show that P(3HB) degrades at a single step and the decomposition peak occurs at approximately 260 °C. The weight loss of the composites is gradual, and the remaining material is increasing with increasing GnP concentration.

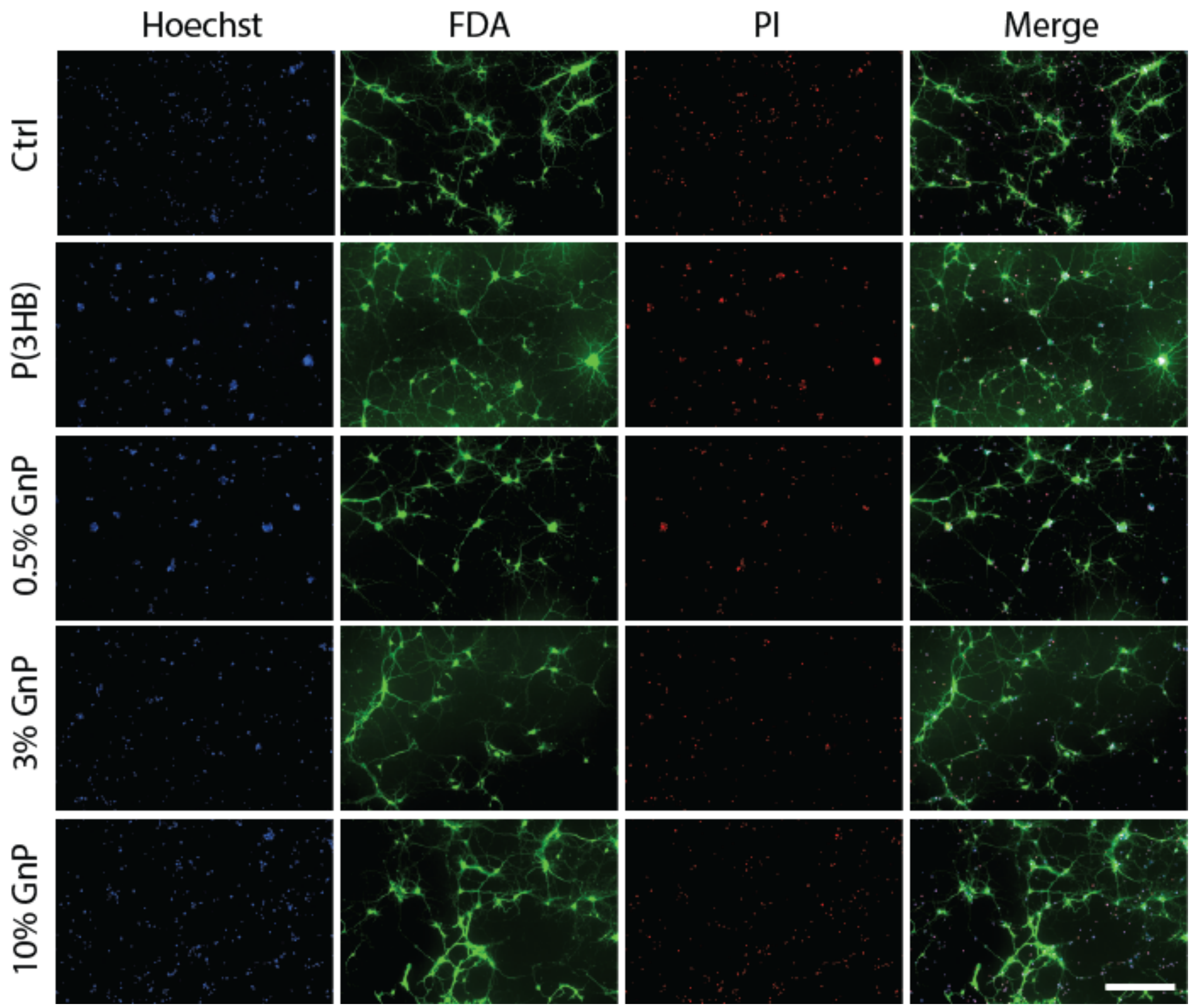

Figure S2: Viability of primary neurons grown onto P(3HB)/GnP composites. Cortical mouse neurons were cultured on P(3HB)/GnP composites (GnP concentrations 0.5, 3 and 10%) for 14 days. Representative images of a neuronal culture stained with Hoechst-33342 (blue) for nuclei visualization, fluorescein diacetate (FDA, in green) for cell viability and propidium iodide (PI) for dead cell visualization (scale bar: 200 μm).

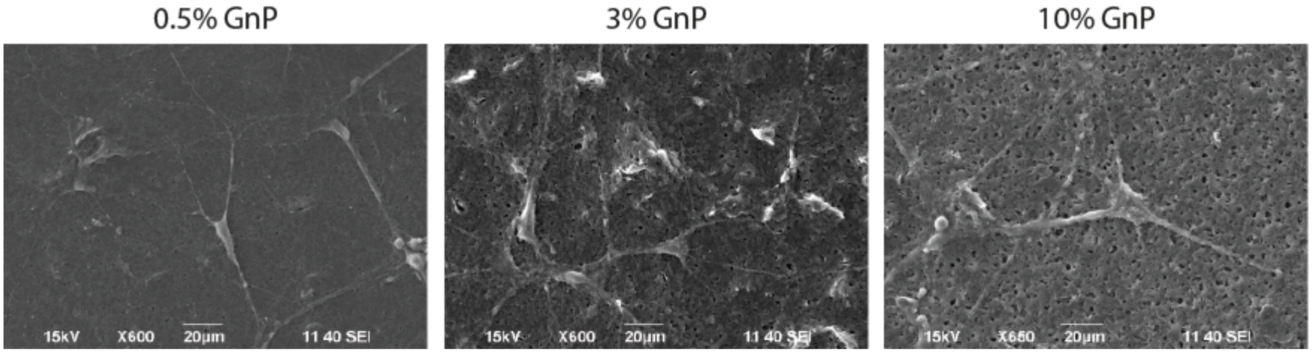

Figure S3. SEM micrographs of neuronal networks grown onto P(3HB)/GnP composite islands.

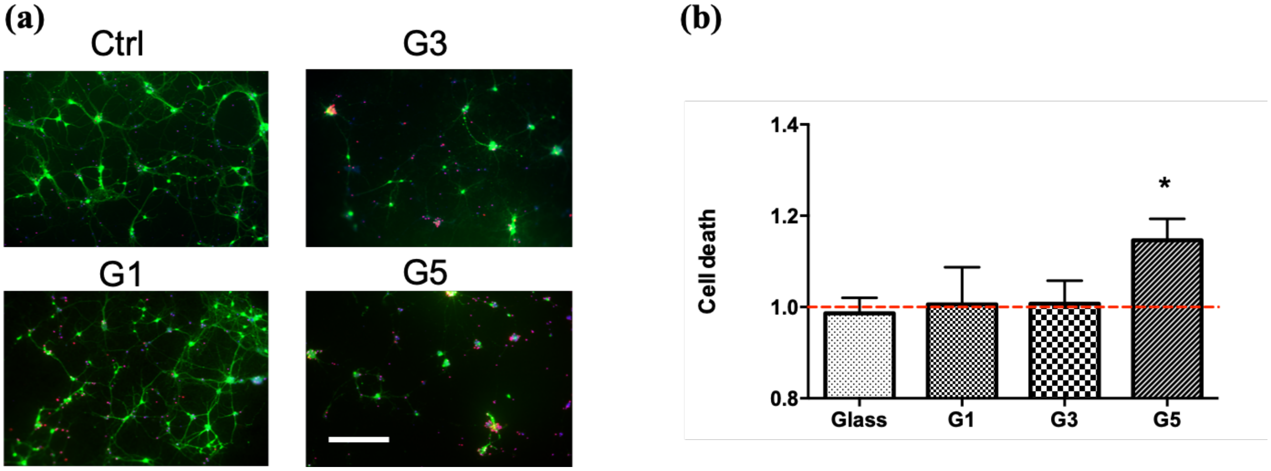

Figure S4: Neuronal viability on free standing P(3HB)/GnP substrates. Cortical mouse neurons were cultured on free standing P(3HB)/GnP films (GnP concentrations 1, 3 and 5 wt%) for 14 days. **(A)** Representative images of a neuronal culture stained with Hoechst-33342 (blue) for nuclei visualization, fluorescein diacetate (FDA, in green) for cell viability and propidium iodide (PI) for dead cell visualization (scale bar: 200 µm). **(B)** Cell viability, evaluated by fluorescence microscopy, is shown. The percentages of PI-positive cells with respect to the total number of Hoechst-positive cells, calculated for each experimental group, were normalized to the values of glass substrate set to 1. A significant increase in cell death was reported for neurons grown onto free standing 5% GnP films. Data are expressed as means ± sem. One-way ANOVA/Bonferroni's tests, n=7 fields per experimental condition, from 2 independent neuronal preparations.

The morphology of the neuronal network investigated by epifluorescence microscopy and presented in **Figure S4a** shows the presence of several neuronal clusters. As displayed in **Figure S4b**, no evident cytotoxic effects were reported in neurons grown onto free-standing films when compared to neurons plated onto pure P(3HB), confirming the good compatibility of the substrates, except for the free-standing film at 5% of graphene.

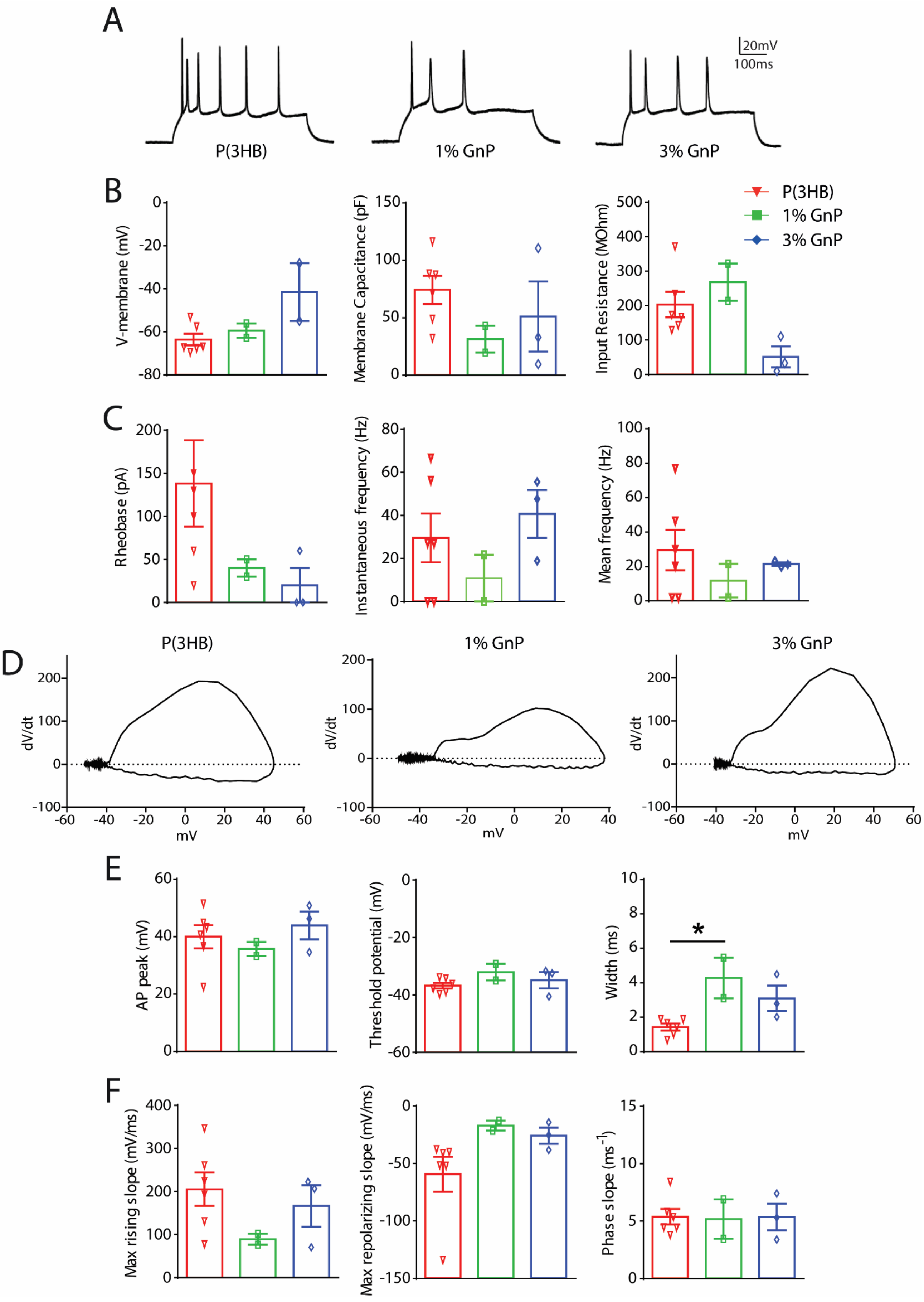
A
20mV
100ms
P(3HB)
1% GnP
3% GnP
B
V-membrane (mV)
Membrane Capacitance (pF)
Input Resistance (MOhm)
C
Rheobase (pA)
Instantaneous frequency (Hz)
Mean frequency (Hz)
D
dV/dt
mV
E
AP peak (mV)
Threshold potential (mV)
Width (ms)
*
F
Max rising slope (mV/ms)
Max repolarizing slope (mV/ms)
Phase slope (ms-1)

Figure S5: Passive and active properties of primary neurons grown onto free standing P(3HB)/GnP composite films. (A) Representative traces of AP firing evoked by 250 pA current injection. (B) From left to right: resting membrane potential (V-membrane), membrane capacitance and input resistance. (C) From left to right: Rheobase, instantaneous firing frequency (left) and mean firing frequency (right) evoked by injection of 250 pA depolarizing current. (D) Representative phase-plot analysis of the AP waveform under each experimental condition obtained by plotting the first derivative of the membrane voltage (dV/dt) versus membrane voltage (Vm). (E) From left to right: AP peak, threshold potential and width. (F) From left to right: max rising slope, max repolarizing slop and phase slope. All data are shown as means ± sem with individual experimental points. Kruskal-Wallis test and Dunn's post-hoc test; n=6, 3 and 2 for P(3HB), 0.5 and 3 GnP, respectively).

Because of the graphene flakes opacity, it was extremely challenging to see cells at the electrophysiology setup optical microscope. To avoid this problem, we treated neurons with FDA (labelling living cells) and filled the recording pipet with fluorescein. However, due to the cell-clumping, proper recording was not possible. As reported in **Figure S5b-c** and **S5e-f**, we were able to obtain results from only few cells. No significant changes were observed in terms of passive and active firing properties. It is important to note that, due to the small number of cells that could be recorded, we cannot formulate any direct conclusions.
